# Snow leopards, prey, and pastoralists: Understanding the impacts of climate change on human–wildlife coexistence in Central Asia

**DOI:** 10.1007/s13280-025-02321-7

**Published:** 2025-12-07

**Authors:** Arash Ghoddousi, Juliana Eggers, Katrin Kirchner, Lydia Cornu, Ismoil Kholmatov, Zairbek Kubanychbekov, Mirzo N. Mirzoev, Kenzhekan Sultanbaeva, Kubanychbek Zhumabai Uulu, Matthias Baumann, Stefan Michel, Tatjana Rosen, Koustubh Sharma, Maarten Hofman, Tobias Kuemmerle

**Affiliations:** 1 Geography Department, Humboldt-University, Berlin, Germany; 2 Wildlife Ecology and Conservation Group, Wageningen University and Research, Droevendaalsesteeg 3a, 6708 PB Wageningen, The Netherlands; 3 Ecosystem Dynamics and Forest Management Group, School of Life Sciences, Technical University of Munich, Munich, Germany; 4 Berchtesgaden National Park, Berchtesgaden, Germany; 5 Tajikistan Nature Foundation, Dushanbe, Tajikistan; 6 Tajik National University, Dushanbe, Tajikistan; 7 Ilbirs Foundation, Bishkek, Kyrgyzstan; 8 Snow Leopard Trust, Seattle, USA; 9 Snow Leopard Foundation, Bishkek, Kyrgyzstan; 10 Haina/Nessetal, Germany; 11 Conservation X Labs, Ashgabat, Turkmenistan; 12 Ecosystems Division, United Nations Environment Programme, Vienna, Austria; 13 Integrative Research Institute on Transformations of Human-Environment Systems (IRI THESys), Humboldt-University, Berlin, Germany

**Keywords:** Human–wildlife conflict, Kyrgyzstan, Livestock depredation, Pamir, Social–ecological resilience, Tajikistan

## Abstract

Fostering coexistence between people and wildlife requires understanding their interactions and how they might change in the future. Climate change potentially impacts coexistence by exacerbating resource scarcity, shifting land-use patterns or changing human–wildlife encounters. Assessing challenges and opportunities for coexistence under climate change is, therefore, a conservation priority. We focused on the Central Asian highlands to investigate the spatial interactions between the snow leopard, its prey, and pastoralists and to explore how climate change might affect these interactions. Our results reveal regionally diverging patterns, with a potential for increased human–snow leopard conflict in Tajikistan (4–17% higher distributional overlap), yet an increasing spatial separation of wildlife and pastoralists in Kyrgyzstan (11–26% lower overlap). As a result, even under the same climate change scenario, some landscapes will likely require conflict mitigation measures, while others will offer opportunities for restoring wildlife. This highlights the need for context-specificity to foster positive human–wildlife interactions under climate change.

**Supplementary Information:**

The online version contains supplementary material available at 10.1007/s13280-025-02321-7.

## Introduction

Human–wildlife coexistence—defined as a dynamic but sustainable process of coadaptation in landscapes where people and wildlife share limited resources—is increasingly desired in the Anthropocene, yet is often hard to achieve (Carter and Linnell 2016; Glikman et al. 2021; Thapa et al. 2024). This is mainly due to the complexity of perspectives about what would determine coexistence, often depending on the varying contexts and needs of stakeholders (Glikman et al. 2021). A considerable body of research now points to the importance of considering social–ecological context for fostering coexistence. Key factors include landscape structure, land-use practices, resource availability for wildlife, human perception of wildlife, and institutions governing natural resource use (Bruskotter et al. 2022; Gao and Clark 2024). Many of these conditions will change in the future as climate change unfolds, likely altering human–wildlife interactions, for example, by increasing spatial overlap between them where land use expands or contracts, or where species’ ranges shift as they track suitable habitat (Abrahms et al. 2023; Newsom et al. 2023; Ma et al. 2024). However, what is less clear is how changes in these conditions might affect coexistence.

Coexistence does not imply the absence of conflict, and it can take many forms (Hill 2021). As a dynamic process, it can persist despite disturbances if conditions supporting both people and wildlife are maintained. This capacity to withstand change or recover from it without shifting to a qualitatively different state is central to the concept of resilience (Folke et al. 2010). A resilience framework thus offers a lens to examine how human–wildlife interactions evolve in response to global change. It can help assess whether these changes support the conditions needed to sustain coexistence or whether they disrupt those conditions, causing its breakdown. Resilience, however, is not inherently beneficial for conservation and those stakeholders trying to achieve conservation goals. Systems characterized by high levels of conflict, or even species loss, may be highly resilient to change, just as those supporting coexistence may prove fragile (Angeler and Allen 2016; Carter and Linnell 2023). Understanding how shared landscapes may change, and what opportunities these changes create for maintaining or transforming coexistence states, is therefore, a key priority.

Coexistence is a context-dependent process, influenced by shifting social values, governance arrangements, and patterns of resource use (Gao and Clark 2024). A recently developed coexistence framework provides a tool to conceptualize and characterize existing coexistence states (Carter and Linnell 2023). These states are not static categories but rather the outcome of the interaction between social and ecological factors within the system. Such factors include human tolerance, institutional capacity, and wildlife adaptability. The framework distinguishes eight coexistence states based on the degree of coadaptation and system resilience. Four of these (i.e., sustained co-benefits, conservation reliance, tolerant synanthropy, and fragile stability) are consistent with coexistence. The other four (i.e., reciprocal damages, eradication, sporadic nuisance, and zero-sum losers) are not (Carter and Linnell 2023) (Online Resource Table S1). Identifying a coexistence state based on this framework could provide a baseline for exploring the evolution of that system over time, potentially informing adaptive conservation strategies aimed at transitioning to more desirable coexistence states. For example, in Sri Lanka, traditional crop-guarding techniques, such as communal night watches and deterrents, have been replaced in some areas by fortified fencing, shifting human–elephant interactions from coexistence to spatial separation (Fernando et al. 2005). Similarly, some ranchers in the USA have transitioned from predator eradication to non-lethal strategies, fostering a shift from conflict-driven management to more stable coexistence with large carnivores (Stone et al. 2017). Thus, the framework by Cater and Linnell (2023) could help to explore the resilience of human–wildlife coexistence under global change but to our knowledge, no study has done this so far.

Climate change, especially in mountainous landscapes, can be a major driver of social–ecological change and, consequently, human–wildlife interactions (Alexander et al. 2015; Urban 2018; Abrahms et al. 2023). Rising temperatures and shifting precipitation patterns, and through these, changes in vegetation and wildlife distributions, can impact human–wildlife interactions by exacerbating resource scarcity or altering predator–prey interactions (Towns et al. 2009; Rabaiotti et al. 2021; Vargas et al. 2021). In mountainous landscapes, climate and vegetation changes also affect pastoralist communities that rely on limited pastures for livestock grazing, forcing or enabling them to spatially or temporally shift grazing patterns, potentially encroaching on wildlife habitats (Dong et al. 2011; Nettier et al. 2017), increasing competition between livestock and wild herbivores or leading to rising conflict with predators due to livestock depredation (Alexander et al. 2015; Abrahms et al. 2023). These changes could undermine coexistence states and intensify conservation challenges (Newsom et al. 2023). Conversely, these changes could lead to spatial separation of people and wildlife or increase resource availability, which would reinforce or open up possibilities for coexistence (Xiao et al. 2022).

Central Asian mountains and their biodiversity are particularly sensitive to the impacts of global change (Yu et al. 2021), providing an important case study for exploring human–wildlife coexistence. This region supports a century-long relationship between the snow leopard (*Panthera uncia*), mountain ungulates as its main prey, and pastoralist communities. Climate- and land-use changes could, however, alter the balance of this relationship, potentially leading to changes that affect coexistence (Aryal et al. 2014b; Young et al. 2024). In this study, with the goal of exploring future potential for human–wildlife coexistence, we assessed possible human–wildlife interactions in the highlands of Central Asia under different scenarios of climate change for 2050 and 2070. Specifically, we used species distribution modeling to approximate current and future distributions of the snow leopard, two of its main prey species (argali *Ovis ammon* and Asiatic ibex *Capra sibirica*), and livestock pastoralists in two regions in Kyrgyzstan and Tajikistan. We assumed an increase in the future spatial overlap between snow leopards and livestock would increase potential livestock depredation (Aryal et al. 2014a, b; Wang et al. 2024). Similarly, we assumed an increase in the future spatial overlap between mountain ungulates and livestock would increase potential competition over forage and space, indirectly impacting the snow leopard through prey depletion (Suryawanshi et al. 2017). Both would thus reduce the potential for human–wildlife coexistence. Methodologically, we used a transdisciplinary approach, combining spatial modeling with the expertise of local and international conservation scientists and practitioners, as well as the local people, to answer the following research questions:

- What are plausible current and future distributions of the snow leopard, its prey, and livestock pastoralists under different scenarios of climate change?
- What are trends in spatial overlaps of the snow leopard, its prey, and livestock pastoralists?
- How could changes in spatial overlaps potentially shift coexistence states?

## Materials and methods

### Study area

**Fig. 1:**
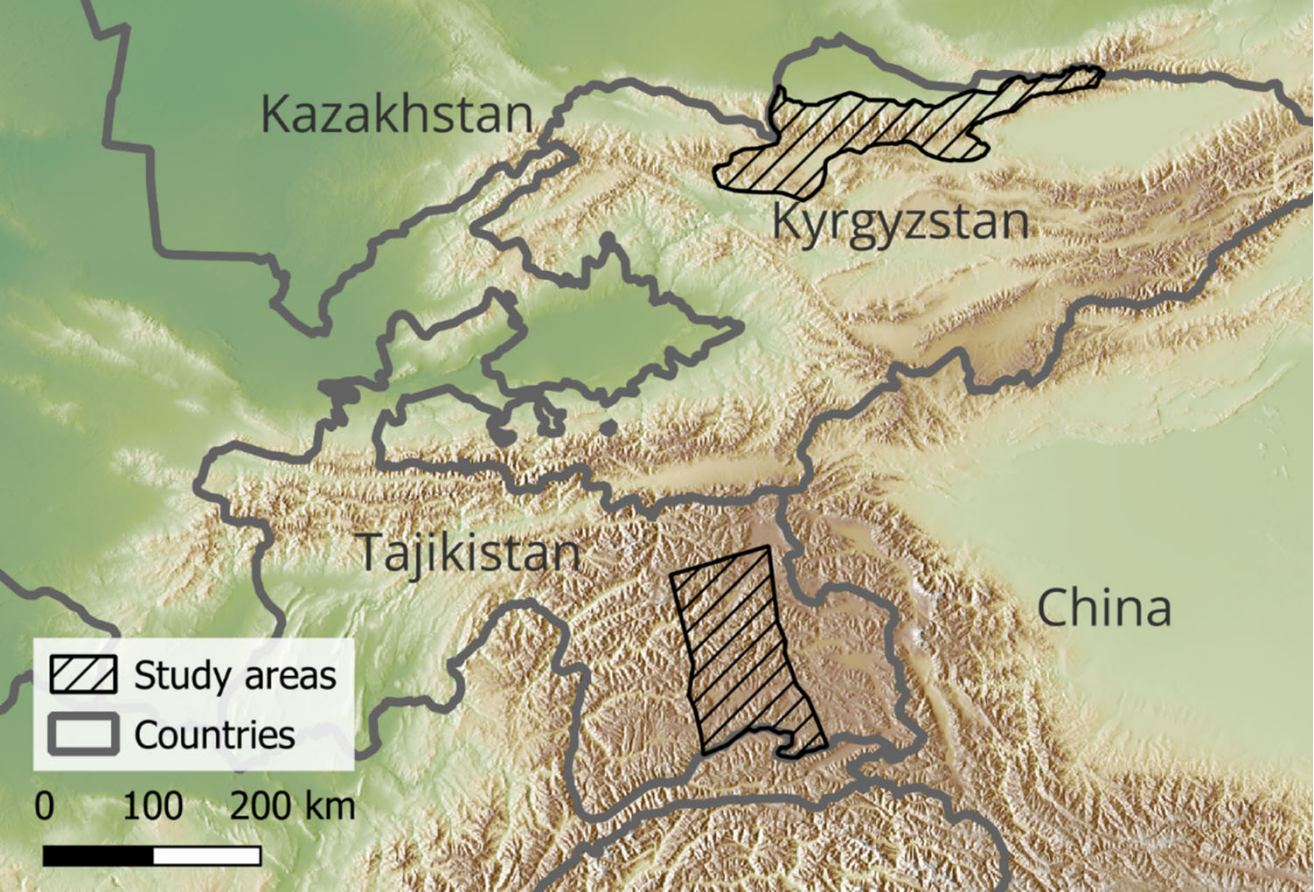
Study landscapes (hashed areas) in Kyrgyzstan and Tajikistan in Central Asia

Our study focused on two large landscapes in Central Asia (Fig. 1). One is located in northern Kyrgyzstan (13 050 km^2^), covering large sections of the Kyrgyz Alatoo Range and adjacent mountains. The elevation range is from 708 to 4839 m a.s.l. It includes Chong-Kemin State Nature Park (IUCN category II) and Ala Archa State Nature Park (cat. II) as well as Shumkar-Tor Conservancy, Shamshy Conservancy, and the summer pastures in Suusamyr. Pastoralism is the main land use in this area but recently, nature-based tourism has become a common activity in this landscape. The area has cold winters, warm summers, and an average annual precipitation of 220 mm. This rugged terrain leads to high climatic diversity, which is also expressed in differences in the vegetation. Typically, northern slopes are forested with Tien Shan spruce (*Picea schrenkiana*) and juniper (*Juniperus sp.*) due to higher precipitation, while more arid southern slopes are covered by mountain steppe vegetation. The Suusamyr Valley is covered by alpine meadows and mountain steppe. In Kyrgyzstan, communities show moderate tolerance for snow leopard, which is attributable to the national pride in snow leopard and awareness campaigns. There are active pasture communities, and pilot conflict mitigation measures are in place, although governance capacity is unevenly distributed (Skrimizea et al. 2022).

The second landscape is located in eastern Tajikistan (16 347 km^2^), in the high plateau of the Eastern Pamir and the transitional areas toward the Western Pamir. The elevation ranges from 2891 to 6154 m a.s.l. It includes the Zorkul Strict Nature Reserve (cat. Ia) and parts of Tajik National Park (cat. II), the southern slopes of the Northern Alichur Mountain Range, and the upper reaches of the Bartang Valley. Here also, pastoralism is the main land use but conservancies with the aim of wild ungulate trophy hunting also exist in the landscape. The area receives less than 100 mm of precipitation annually and has mean annual temperatures around 0 °C, with the mean temperature of the coldest month below − 15 °C. Compared to the Kyrgyz landscape, these areas receive much less rainfall, are colder, and have shorter vegetation periods due to their higher elevation. Accordingly, vegetation is scarcer, dominated by small shrubs and spiny cushion plants, and forests are missing. In Tajikistan, key mediating factors for coexistence states are relatively weak governance capacity, including limited investment in pasture management and conservation. Mitigation tools are largely absent, and where frequent livestock losses occur, tolerance for wildlife is low (Skrimizea et al. 2022).

Central Asia is crucial to the conservation of the snow leopard, with an important share of the population thought to be in Kyrgyzstan (~ 150 individuals; 4–5% of the global population) and Tajikistan (250–280 individuals; 7–10% of the global population) (Nowell et al. 2016; Saidov et al. 2016; McCarthy et al. 2017). Snow leopard attacks livestock, either on pastures or inside livestock corrals, where it tends to kill many animals at once. Often, such depredation results in retaliatory killings of snow leopard—a severe threat to the species. For the period 2010–2016, based on snow leopard kill seizures, observations, and market surveys, it was estimated that 5–7 snow leopards in Kyrgyzstan and 20–25 in Tajikistan were poached each year (Nowell et al. 2016). The main prey species of the snow leopard in the Tajik landscape are argali and Asiatic ibex, while in the Kyrgyz landscape, only Asiatic ibex occurs. Marmots (*Marmota sp*.) are also important prey for snow leopards (Jumabay-Uulu et al. 2014) but we did not include them in this study due to a lack of data.

### Overall conceptual and methodological frameworks

**Fig. 2:**
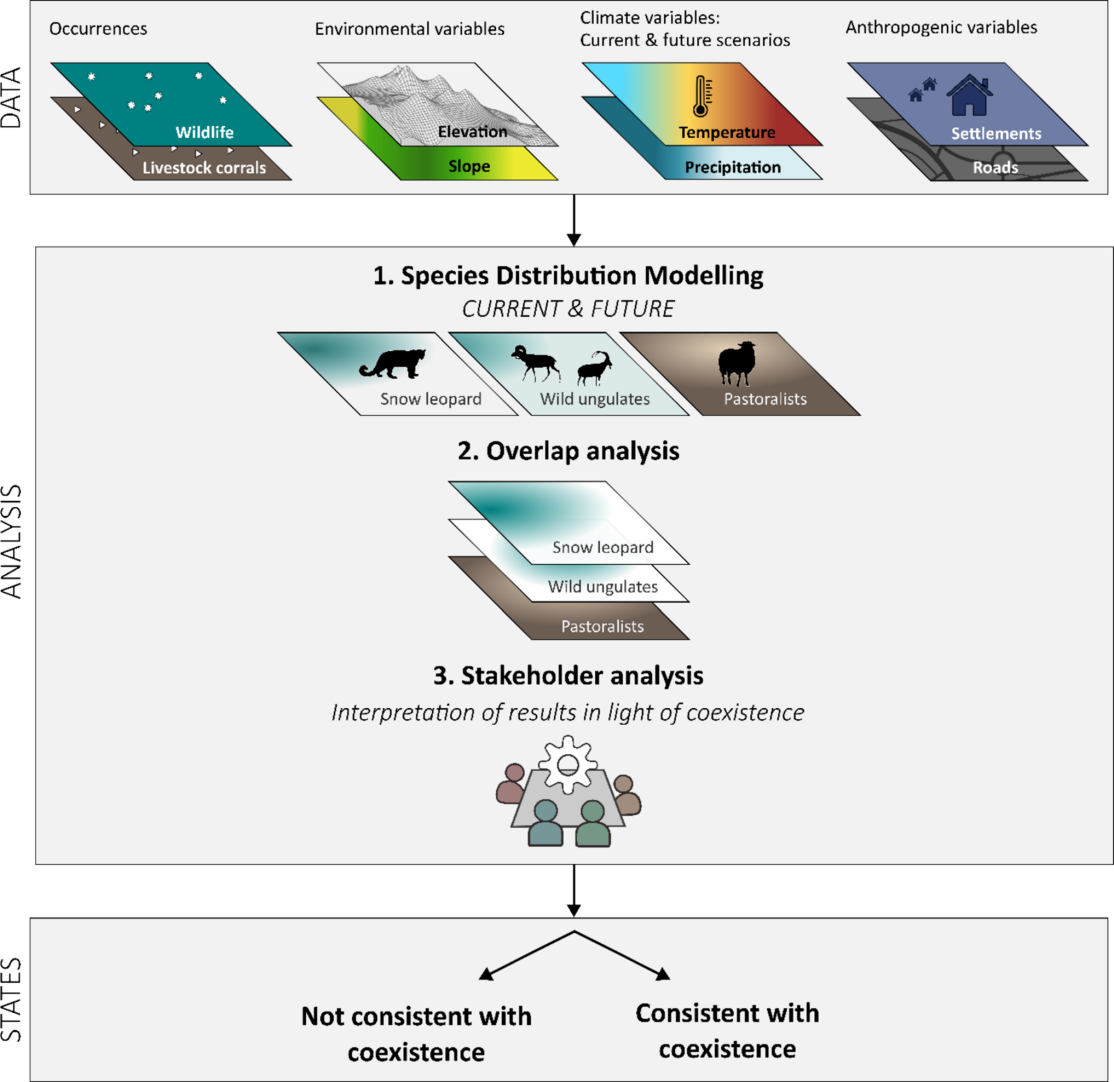
Methodological framework to assess human–wildlife coexistence states under future climate scenarios in Kyrgyzstan and Tajikistan

We used a transdisciplinary approach to assess the current and future potentials for coexistence between people and wildlife in our study landscapes (Fig. 2). We began by exploring the potential pathways through which climate change may influence human–wildlife interactions. To do so, we synthesized information from the scientific literature, technical reports, stakeholder workshops, and local experts. This allowed us to identify key social–ecological interactions shaping coexistence, including both the direct effects of climate change on snow leopards and indirect effects through prey and habitat-mediated changes. Second, we mapped the current distribution of the snow leopard, its prey, and pastoralists. We then projected these distributions into the future and assessed the trends in spatial overlaps. Finally, we built on Carter and Linnell’s framework (2023) to assess within stakeholder workshops how changes in these interactions, approximated via changes in range overlap between snow leopard, its prey, and pastoralists, may alter coexistence states (Online Resource Table S1), either by reinforcing existing adaptation strategies or leading to increased competition and conflict. Thus, rather than directly measuring resilience, we used resilience as a conceptual lens to explore whether and how current human–wildlife interactions may be maintained or disrupted under future pressures.

### Potential impacts of climate change on coexistence

**Fig. 3:**
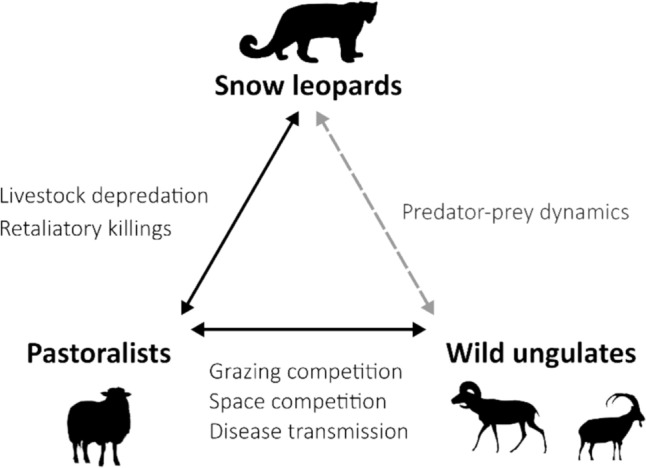
The relationships between the snow leopard, its prey, and the pastoralists in Kyrgyzstan and Tajikistan. Since the relationship between the snow leopard and wild ungulates does not directly impact coexistence, we represent it here with a dashed line

We used a combination of expert knowledge and stakeholder participation in transdisciplinary workshops (two online workshops with participation of 9 and 11 local and international conservation scientists and practitioners with experience from the study landscapes) as well as the scientific literature (Xiao et al. 2022; Wang et al. 2024), climate predictions (Forrest et al. 2012; Li et al. 2016; Yu et al. 2021), and technical reports (Skrimizea et al. 2022) to identify pathways of potential climate change impacts on human–wildlife coexistence (Fig. 3). Although snow leopard is adapted to some of the harshest environments on the planet, the direct effects of climate change on its physiology is still largely unknown. Climate models predict warmer temperatures and increased aridity in the summer alongside greater precipitation in spring and autumn in Central Asia (Aalto et al. 2017; Skrimizea et al. 2022). This is likely to modify the composition and phenology of vegetation in the snow leopard habitat (Forrest et al. 2012), which would affect the density and distribution of mountain ungulates (Salas et al. 2020; Xiao et al. 2022; Wang et al. 2024). This could then potentially affect the snow leopard through modified prey availability (Kitchener et al. 2024). If these changes lead to lower prey availability, they may intensify the probability of livestock depredation (Khorozyan et al. 2015a). Moreover, changes in vegetation have implications for the distribution of pastoralists who compete with wild ungulates for forage and space (Forrest et al. 2012; Ekernas et al. 2017). Warming might either allow local people to use previously inaccessible pastures (e.g., at higher elevations) or alternatively, due to aridification, lead to the abandonment of rangelands (Li et al. 2016). These changes could lead to variations in the contact zones between domestic and wild herbivores, and thus, competition between these two, or the risk of disease transmissions (Khorozyan et al. 2015b; Ekernas et al. 2017). While an increasing spatial overlap of livestock and snow leopard could intensify depredation events, leading to increased retaliatory killing of snow leopard (Aryal et al. 2014b; Rabaiotti et al. 2021; Wang et al. 2024), a reduced spatial overlap may lead to opportunities for coexistence. We used this conceptual framework (Fig. 3) to guide our analysis of how climate- and land-use changes may influence coexistence in our study system.

### Current and future distributions of snow leopard and its prey

We used occurrence data mostly collected through camera trapping, direct observations, and field surveys between 2009 and 2022. This included argali (977 occurrences, only from Tajikistan), Asiatic ibex (Kyrgyzstan: 359; Tajikistan: 1456), and snow leopard (Kyrgyzstan: 31; Tajikistan: 83). Argali is considered locally extinct in the study landscape in Kyrgyzstan. Despite the lower number of snow leopard occurrences from Kyrgyzstan, we refrained from using signs (e.g., tracks, scats) in our models as there is a higher likelihood of misidentification in such data. As some of our wild ungulate data come from winter surveys, conducted primarily using vehicles, we removed all occurrences within the 10% percentile of distance from roads to lower potential spatial bias (Kadmon et al. 2004). To lower spatial bias and avoid spatial autocorrelation, we also thinned our occurrences to a minimum distance of 500 m between them.

We prepared a set of environmental variables (Table 1) for our models, ensuring that they were all in the same spatial resolution (250 m) and projection (WGS84 EPSG:3857). We only used variables that were available for the current and future, and used only variables deemed ecologically relevant for the specific species. We scaled all variables and ran correlation tests (variation inflation factor, Spearman’s rank) to avoid using highly correlated variables. From each pair of highly correlated variables, we retained the one with the most ecological relevance to the target species or the one with the highest contribution to the model. We used two topography variables (elevation, ruggedness), two anthropogenic variables (distance to roads, distance to settlements), and 10 climate variables based on the CHELSA climatologies at 1-km resolution (averaged values for the period 1979–2013). Regarding future climate, we used data for two ‘shared socioeconomic pathway’ scenarios (SSP2-4.5 and SSP5-8.5) for two time steps (2050 and 2070). SSP2-4.5 represents a ‘Middle of the Road’ development path in which economic and technological growth proceeds relatively smoothly and global cooperation on sustainability issues remains limited. This scenario assumes that some climate protection measures will be taken. SSP5-8.5 represents the most drastic climate impacts forecasted in any of the scenarios (Riahi et al. 2017).

**Table 1:** Topographic, anthropogenic, and climatic variables in the species distribution models of the snow leopard, its prey, and the pastoralists in Tajikistan and Kyrgyzstan. *A* argali*, AI* Asiatic ibex*, L* livestock*, SL* snow leopard

Category	Variable	Species	Unit	Source
*Topography*	Elevation	SL, AI, A, L	m	Shuttle Radar Topography Mission (http://search.earthdata.nasa.gov)
	Ruggedness (standard deviation of slope)	SL, AI, A, L	Standard deviation	Shuttle Radar Topography Mission (http://search.earthdata.nasa.gov)
*Anthropogenic*	Distance to settlements	SL, AI, A, L	m	Open Street Map (www.openstreetmap.org)
	Distance to roads	SL, AI, A, L	m	Open Street Map (www.openstreetmap.org)
*Climatic*	Annual mean temperature (bio1)	AI, A, L	°C	CHELSA (https://chelsa-climate.org/)
	Temperature seasonality (bio4)	SL, AI, A, L		CHELSA (https://chelsa-climate.org/)
	Maximum temperature of warmest month (bio5)	SL	°C	CHELSA (https://chelsa-climate.org/)
	Minimum temperature of coldest month (bio6)	SL, AI, A, L	°C	CHELSA (https://chelsa-climate.org/)
	Mean temperature of warmest quarter (bio10)	SL	°C	CHELSA (https://chelsa-climate.org/)
	Mean temperature of coldest quarter (bio11)	AI, A, L	°C	CHELSA (https://chelsa-climate.org/)
	Annual precipitation (bio12)	SL, AI, A, L	mm/year	CHELSA (https://chelsa-climate.org/)
	Precipitation of wettest month (bio13)	SL, AI, A, L	mm/month	CHELSA (https://chelsa-climate.org/)
	Precipitation seasonality (bio15)	SL	Coefficient of variation	CHELSA (https://chelsa-climate.org/)
	Precipitation of wettest quarter (bio16)	AI, A, L	mm/quarter	CHELSA (https://chelsa-climate.org/)

As our species distribution modeling algorithms, we used maximum entropy (Maxent), random forest (RF), and boosted regression trees (BRT) in an ensemble framework in the *biomod2* package (Guéguen et al. 2025) in R. Our algorithms require pseudo-absences (RF and BRT) or background points (Maxent). We generated pseudo-absences to be ten times the occurrences for each species, and for background points, we used the default 10,000 points. We restricted the pseudo-absences and background points to a minimum of 250 m and a maximum of 50 km distance around occurrence points. We ran separate models for each species, study landscape, climate change scenario, and time step, resulting in a total of 25 ensemble models (Kyrgyzstan: 10 and Tajikistan: 15). For RF and BRT, we used 1000 trees each, and for RF, our sample size was 250. We created the ensemble models with the weighted mean of all models involved. We performed cross-validation with two model runs and by splitting the data into training (80%) and test (20%) datasets. We assessed model performance in each case using (1) model statistics (e.g., the area under the curve AUC of receiver operating characteristic ROC), (2) the response curve of environmental, anthropogenic, and climatic variables and whether the predictions are plausible, and finally (3) cross-checking the predicted maps with local experts in an online workshop for each study landscape (with 5–6 participants) to validate and fine-tune model outputs. We predicted the best-performing models with a 150 km buffer around the study landscape to avoid boundary effects. However, we only considered the study landscapes in each country in our subsequent analyses.

To translate the continuous habitat suitability predictions to binary (i.e., habitat vs. non-habitat) maps, we used the maxSSS threshold (i.e., maximum value of model sensitivity plus specificity) (Liu et al. 2005). We then merged patches that were < 1 km apart from each other into multi-part polygons because such patches were well within the distances normally traveled by these species. Finally, we removed habitat patches that were smaller than the minimum home range size of the species (i.e., argali: 76 km^2^, Asiatic ibex: 40 km^2^, snow leopard: 154 km^2^), based on minimum home range estimates from the literature or previous studies in Central Asia (Reading et al. 2003, 2007; Davletbakov et al. 2024).

### Current and future livestock distribution

To map livestock grazing pressure across both study landscapes, we combined interviews and satellite data. First, we conducted interviews with pastoralists (Kyrgyzstan: 86, Tajikistan: 54) to gather spatially explicit locations of corrals for the year 2020, as well as information on livestock types (i.e., cattle, yak, horse, sheep, and goat) and numbers, the extent of respective pasture, and cases of conflict with large carnivores. For the Kyrgyz landscape, we had 171 corral locations available, mostly in the Chon-Kemin Valley. For the Tajik landscape, we had a total of 46 corral locations available, mostly from the Murghob District. As livestock corrals are more widespread, we used high-resolution satellite images available on Google Earth^1^ to identify additional corrals. Corrals can be identified as artificial structures (e.g., stone walls) in open pastures with trampled open soil surrounding them (Bleyhl et al. 2019; Ghoddousi et al. 2024). We used the combined corral location dataset (Kyrgyzstan: 743 and Tajikistan: 356) as occurrences in species distribution models, using the same ensemble approach as for the wildlife species, to assess the current and future distribution of areas suitable for livestock pastoralism. We added a 5-km buffer around identified patches, informed by the interview responses, as livestock typically move within a radius around corrals (McGranahan et al. 2018). We also removed areas with slopes steeper than 45 degrees and overlapping bodies of water from the predicted areas used by livestock.

### Current and future coexistence states

We calculated the habitat patch area per landscape, species, and climate scenario for each time step and calculated relative habitat gain and loss compared to current conditions. Moreover, we created habitat overlap maps and calculated the area and percentage of overlap gain and loss compared to current conditions for: (a) snow leopard–livestock (signaling conflict); (b) wild ungulates–livestock (competition); and (c) snow leopard–prey (prey availability). Finally, we identified the areas of potential livestock depredation or competition between livestock and wild ungulates based on the overlap areas, which we interpret as spatial indicators of ecological potential for conflict or competition, rather than direct evidence of human–wildlife interactions. These areas are potential focus areas for proactive conflict mitigation measures (e.g., predator-proof corrals, compensation schemes) and coexistence-oriented pasture management (e.g., reserving parts of pastures for wild ungulates).

To assess current and future coexistence, we examined the three-way relationship (Fig. 3) between the snow leopard, its prey, and pastoralists. First, we worked with stakeholders in two country-specific online workshops (each with participation of 8–9 stakeholders) to characterize the present-day coexistence state (Carter and Linnell 2023). We selected the participants to represent diverse expertise (e.g., wildlife ecology, pastoralism, conservation practice, and governance). We structured the workshops around group discussions guided by model outputs, interview results, technical reports (Skrimizea et al. 2022), and expert knowledge of human–snow leopard interactions and wild ungulate–livestock dynamics. We used facilitated consensus-building: After presenting the available information, participants discussed and jointly agreed on the classification of current coexistence states. Where opinions diverged, consensus was reached through moderated deliberation. We consolidated the workshop outcomes as expert opinion informed by both stakeholder knowledge and supporting reports. Then, we inferred future coexistence states based on changes in spatial overlaps as a proxy for potential competition, depredation risk, and conflict (see above), as well as the socioeconomic and political mediating factors such as governance capacity, institutional trust, adaptive practices, and retaliatory behavior, identified through stakeholder workshops and technical reports (Online Resource Table S2).

## Results

### Current distribution patterns

All models and algorithms performed well (AUC of snow leopard models: 0.84–0.87; Asiatic ibex: 0.78–0.92; and argali: 0.82–0.89). The snow leopard models for Tajikistan predicted annual precipitation as the most important variable (all algorithms), with a positive relationship with habitat suitability. Moreover, maximum temperature of the warmest month was important in RF and BRT with a positive influence. When filtering patches smaller than the minimum home range of the species, most of the study landscape was identified as habitat patches apart from flat areas (Online Resource Fig. S1). The Kyrgyzstan models predicted the most important variables as annual precipitation, precipitation seasonality, and minimum temperature of the coldest month. The predicted snow leopard habitat was small and fragmented, and concentrated in the eastern and central parts of the study landscape (Online Resource Fig. S2).

The most important variables predicting argali habitat suitability in Tajikistan were precipitation of wettest quarter (Maxent and RF), precipitation of wettest month (RF and BRT), annual precipitation (Maxent and RF), and mean temperature of coldest quarter (Maxent and BRT). The predicted argali habitat was mainly distributed on the high plateaus in the eastern and southeastern parts of the study landscape, avoiding high mountains and the bottom of valleys (Online Resource Fig. S3). The most important variables for the Asiatic ibex models in Tajikistan were temperature seasonality (all algorithms), annual mean temperature (RF and BRT), and distance to settlements (RF and BRT). The models predicted ibex habitat in Tajikistan to be more widespread than argali habitat and to occupy most of the mountainous landscapes, apart from the highest peaks (Online Resource Fig. S4). For Kyrgyzstan, annual mean temperature (Maxent and RF), distance to roads (RF and BRT), and elevation (RF and BRT) were the most important variables for predicting ibex habitat. Suitable habitat occurred across most of the mountains of the study landscape but was absent from valley bottoms and flat areas as well as the highest peaks (Online Resource Fig. S5).

When using the corral locations as occurrences in the species distribution model, for Tajikistan, our models predicted precipitation of the wettest quarter and temperature seasonality (in all algorithms) as well as elevation (Maxent and BRT) as the most important variables for livestock pastoralism. The predicted distribution of livestock pastoralism was mainly in the eastern and southern parts of the study landscape (Online Resource Fig. S6). In Kyrgyzstan, the most important variables for livestock pastoralism were elevation and temperature seasonality (both in all algorithms) as well as precipitation of wettest month (RF and BRT). Our models predicted suitable areas for livestock pastoralism across the entire study landscape, apart from the highest elevations (Online Resource S7).

### Future changes in the distributions of the snow leopard, its prey, and pastoralism

Our future projections showed that the snow leopard habitat in Tajikistan will mainly increase until 2050 (both under SSP2-4.5 and SSP5-8.5) but then, depending on the climate scenarios, either remain stable (SSP2-4.5) or increase further in major ways (41%, SSP5-8.5) until 2070 (Table 2). For Kyrgyzstan, our predictions showed an opposite trend, with a decline in suitable snow leopard habitat for the SSP2-4.5 scenario in both time steps, whereas the habitat remained stable for 2050 and then declined substantially (49%) by 2070 in the SSP5-8.5 scenario (Table 2). For prey species, future predictions indicated an increase in argali habitat across all scenarios and time steps, with a drastic potential increase (46%) in 2050 under the SSP2-4.5 scenario (Table 2). Ibex habitat was relatively stable across most time steps and scenarios (Table 2), with an increase in Tajikistan under the SSP5-8.5 scenario until 2050. In both countries, the ibex habitat was expected to decrease under the SSP5-8.5 scenario in 2070.

**Table 2:** Changes in suitable habitat for the snow leopard, its prey, and the livestock pastoralists under different climate change scenarios (SSP2-4.5 and SSP5-8.5) and for two time steps (2050 and 2070) in Tajikistan and Kyrgyzstan. Changes in the habitat > 10% are indicated in bold text. The arrows indicate the overall trend, and the dash refers to relative stability compared to current conditions

		*SSP2-4.5 2050*		*SSP2-4.5 2070*		*SSP5-8.5 2050*		*SSP5-8.5 2070*	
		Gain (%)	Loss (%)	Gain (%)	Loss (%)	Gain (%)	Loss (%)	Gain (%)	Loss (%)
*Argali (Tajikistan)*		**19**	2	**14**	4	**46**	6	**15**	6
*Ibex (Tajikistan)*		2	2	1	6	**15**	0	2	**13**
*Snow leopard (Tajikistan)*		**27**	6	**10**	**11**	**27**	9	**41**	2
*Livestock (Tajikistan)*		**19**	0	**15**	4	**21**	1	4	**12**
*Ibex (Kyrgyzstan)*		1	5	6	2	5	2	1	**14**
*Snow leopard (Kyrgyzstan)*		3	**12**	4	**13**	1	8	1	**49**
*Livestock (Kyrgyzstan)*		0	**16**	0	8	0	9	0	**25**

The future suitable area for livestock pastoralism also changed differently in the two regions (Table 2). In Tajikistan, the area suitable for livestock pastoralism was predicted to increase under all scenarios and time steps, apart from a predicted decline under the SSP5-8.5 scenario in 2070. The conditions in Kyrgyzstan were different, with a decline in 2050 and a stable state in 2070 under the SSP2-4.5 scenario and a stable habitat in 2050 and a decline in the areas suitable for pastoralism in 2070 under the SSP5-8.5 scenario.

### Future overlaps between the snow leopard, its prey, and pastoralism

**Fig. 4:**
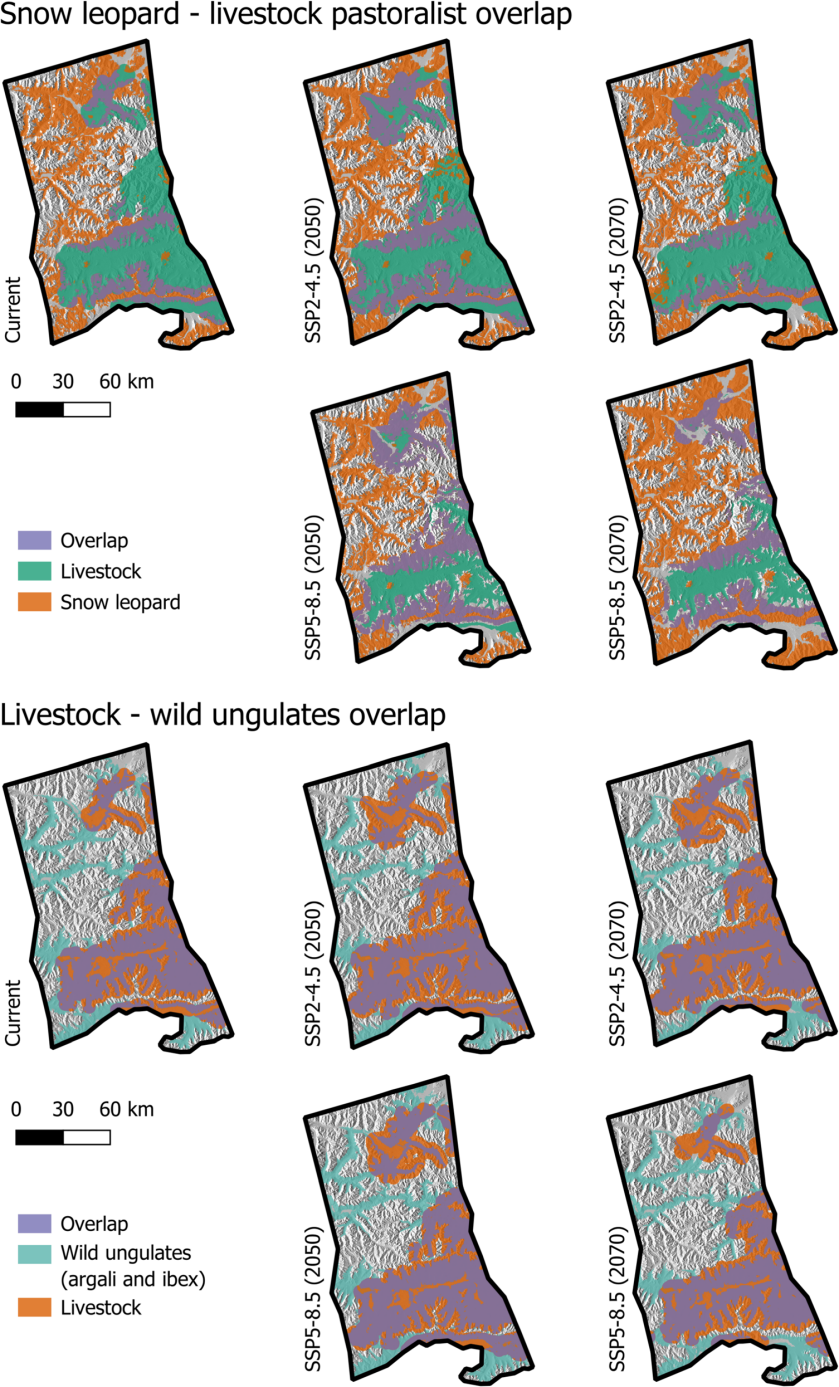
Current and future range overlap of the snow leopard and livestock (left) and wild ungulates and livestock (right) in Tajikistan under different climate scenarios (SSP2-4.5 and SSP5-8.5) and time steps (2050 and 2070)

**Fig. 5:**
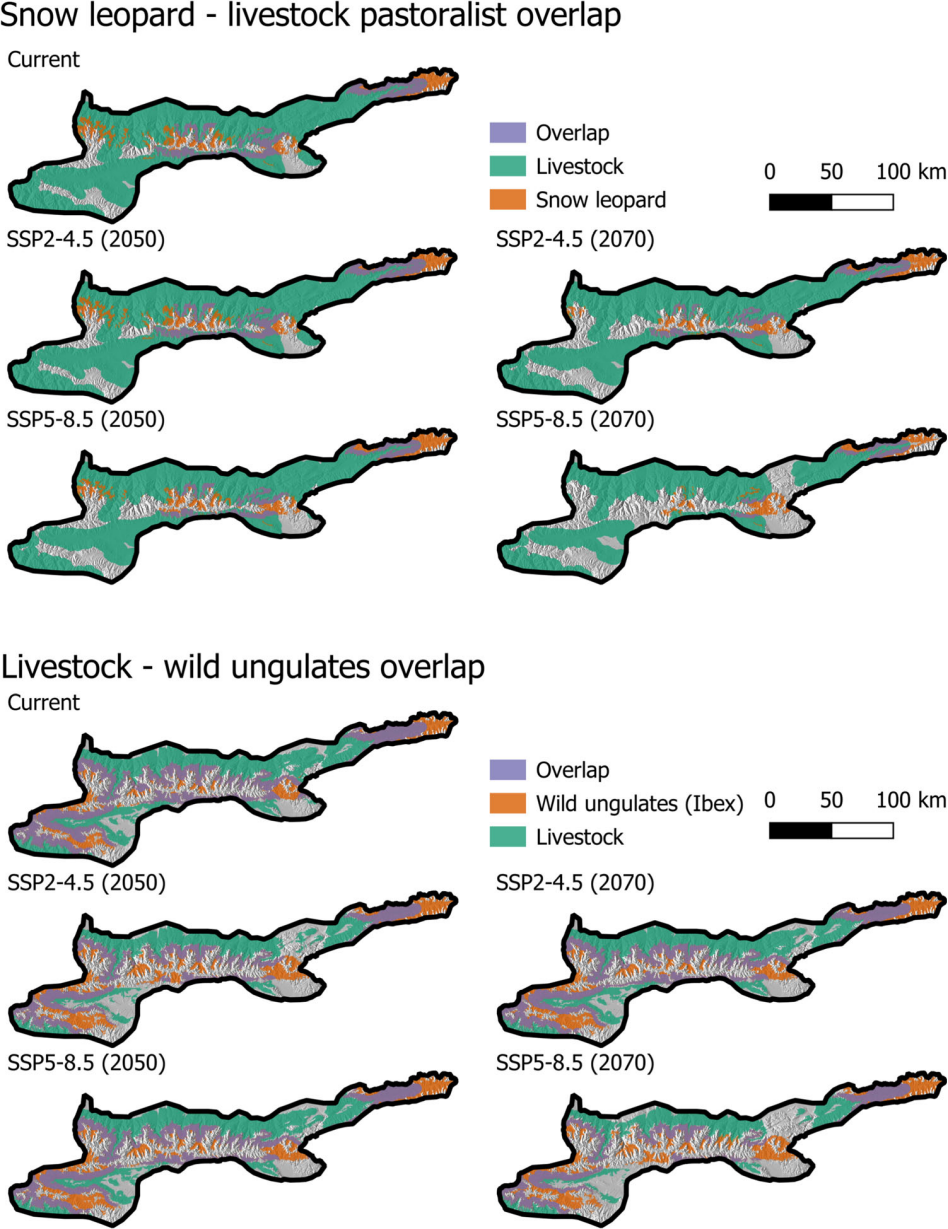
Current and future range overlap of the snow leopard and livestock (up) and wild ungulates and livestock (bottom) in Kyrgyzstan under different climate scenarios (SSP2-4.5 and SSP5-8.5) and time steps (2050 and 2070)

**Fig. 6:**
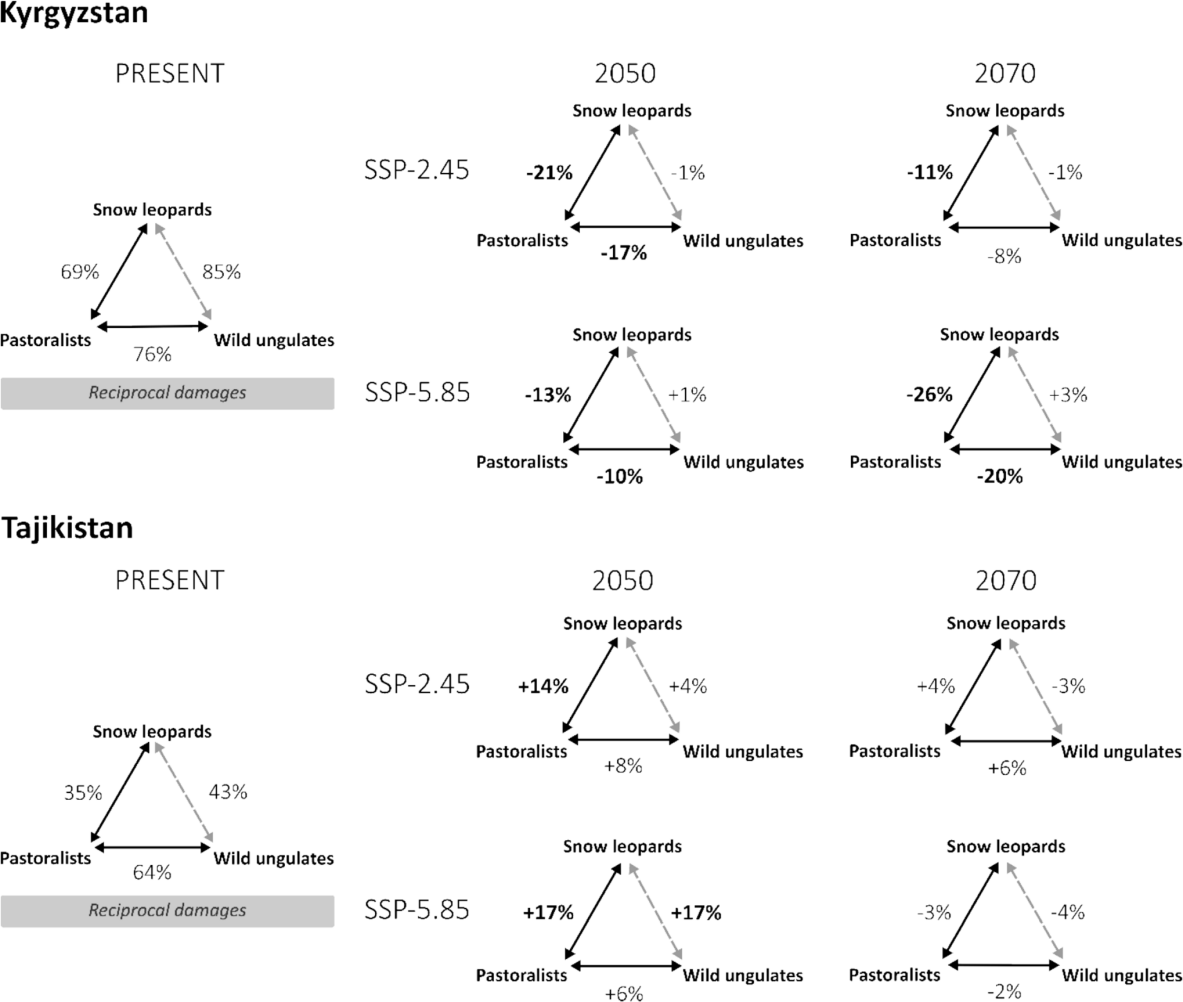
The social–ecological interactions between the snow leopard, its prey, and pastoralists in relation to climate-induced shifts in overlap in Kyrgyzstan (top) and Tajikistan (bottom). The percentage values in the present refer to the amount of overlap in distribution, and the labels refer to coexistence states following Carter and Linnell (2023). The percentage values in future scenarios indicate overlap change (values above 10% in bold font)

Our models predicted an overall increase in the potential human–snow leopard conflict in Tajikistan compared to the current rate, especially for 2050 under both climate scenarios (Figs. 4 and 6). However, this pattern was reversed in Kyrgyzstan, with a medium-to-high decline across all climate scenarios and time steps (Figs. 5 and 6). Potential conflict hot spots, consistently identified across time steps and scenarios, were located in higher elevations, particularly in the northern parts of the Tajik landscape and the eastern parts of the Kyrgyz landscape (Online Resource Fig. S10). These areas should be the focus of proactive mitigation measures.

While the range overlap between wild ungulates and livestock was relatively stable in Tajikistan across all climate scenarios and time steps, there was a medium-to-high decline in the overlap in Kyrgyzstan (Figs. 4, 5, 6). Large areas of potential overlap between livestock and wild ungulates were consistently identified across time steps and scenarios in both countries, which should be prioritized for the implementation of pasture management schemes (Online Resource Fig. S10).

The snow leopard–prey range overlap was relatively stable across different climate scenarios and time steps in both study landscapes (Online Resource Fig. S8 and Fig. S9). Only the overlap under SSP5-8.5 in 2050 in Tajikistan showed an increase.

### Coexistence states

According to our analyses, both study landscapes are currently characterized by relatively high spatial overlap between the snow leopard and pastoralists (Figs. 4 and 5), especially in the Kyrgyzstan landscape, due to the easier accessibility of the terrain and higher productivity of the landscape, more pastoralists are present, resulting in a higher probability of encounters between them and the snow leopard. Moreover, ongoing conflict through livestock depredation and retaliatory killings of the snow leopard is widespread, and negative interactions between wild ungulates and livestock exist (e.g., competition over forage and space, disease transmission). Given this, and after receiving input from various stakeholders in workshops and interviews regarding current human–wildlife interactions, we postulated that both study landscapes are currently in a state of ‘reciprocal damages’ (i.e., frequent encounters prompt strong responses that perpetuate negative impacts on both parties) (Fig. 6) (Carter and Linnell 2023). Despite these conditions, relative stability in species distributions and populations (inferred from local experts and stakeholder workshops) as well as land uses, suggests resilience in both systems.

Under SSP2-4.5 and SSP5-8.5, the potential human–snow leopard overlap, and thus likely also conflict in Tajikistan, was projected to increase substantially by 2050. Given weak governance, limited conflict mitigation schemes, and stable coadaptation capacities, we suggest this indicates a potential worsening of the coexistence state (Fig. 6). However, wild ungulates–livestock and snow leopard–wild ungulates overlaps remain relatively stable (except for a 17% increase in the latter under SSP5-8.5). By 2070, overlaps return to near-present levels under both scenarios, with a 10% decrease under SSP2-4.5 and 20% under SSP5-8.5 (Fig. 6).

In Kyrgyzstan, in 2050, under both SSP2-4.5 and SSP5-8.5, the potential human–snow leopard conflict declines, while the wild ungulates–livestock overlap also decreases under SSP2-4.5. However, the snow leopard–wild ungulates overlap remained stable. Despite moderate governance capacity and limited conflict mitigation schemes, these trends indicate potential improvement in the coexistence state, with resilience hinging on prey availability rather than conflict and competition with livestock (Fig. 6). By 2070, the SSP2-4.5 scenario overlap values remain relatively stable compared to present-day conditions. In contrast, under SSP5-8.5, the continued decline in snow leopard–livestock and wild ungulates–livestock overlaps suggests a decrease in conflict, and therefore, a possible state shift toward a state more consistent with coexistence (Fig. 6).

## Discussion

Climate change is projected to exacerbate human–wildlife relationships (Alexander et al. 2015; Abrahms et al. 2023), especially in mountain landscapes (Urban 2018) but its impacts on coexistence have rarely been considered (Fiasco and Massarella 2022). In this study, we used species distribution models, projected to different climate change scenarios, and explored possible future conditions for human–wildlife coexistence in the mountains of Central Asia. Importantly, we considered livestock pastoralism in current and future scenarios as the most important land use in this region, and how it might spatially overlap with both, snow leopard and its prey. We derived three main findings from our analysis. First, despite major commonalities between our study regions, we identified diverging trends in the future suitable areas for snow leopard, key prey species, and pastoralism, with sharp variations across future climate scenarios. Second, these shifts result in higher overlaps of the snow leopard and its prey distributions with livestock, likely exacerbating human–wildlife conflict and competition between livestock and wild ungulates in Tajikistan, while in Kyrgyzstan, opposite trends are observable. Third, our future projections suggest possible transitions toward worsened states of human–wildlife coexistence in Tajikistan, urging the implementation of conflict mitigation measures. However, the predicted range separation of wildlife and pastoralists in Kyrgyzstan may provide opportunities for restoration. Our findings underscore the need for context-specific conservation strategies under climate change.

Our first primary finding indicated contrasting trends in the snow leopard habitat between the two study landscapes. In Tajikistan, the snow leopard habitat is projected to expand under both climate scenarios, potentially due to increased precipitation and milder temperatures at higher elevations (Skrimizea et al. 2022). In contrast, Kyrgyzstan is expected to experience a contraction in suitable snow leopard habitat, particularly under the SSP5-8.5 scenario by 2070, likely driven by increased aridity that may render parts of the landscape unsuitable for the species. These diverging trends highlight that broad-scale studies might mask important regional variations. For instance, a global study predicted a 23% snow leopard habitat loss associated with climate warming (Li et al. 2016). Similarly, a study suggested that 30% of the snow leopard habitat in the Himalayas may be lost due to a climate-induced shift in the treeline (Forrest et al. 2012). As such, local-scale consequences of climate change for wildlife and people (e.g., variations in available resources or encounters) may be misjudged.

We predicted the distribution of wild prey to remain rather stable, with argali showing a potential habitat expansion across all scenarios and ibex maintaining a largely constant habitat distribution. These results underscore the adaptability of the Asiatic ibex to various climatic conditions (Salas et al. 2020), while showing more favorable conditions for argali in the future. In contrast, livestock pastoralism exhibited substantial variations, increasing in Tajikistan under most scenarios while declining in Kyrgyzstan. This divergence suggests that pastoralists in Tajikistan may expand into higher elevations, whereas those in Kyrgyzstan may face constraints such as declining pasture quality or increased aridity. These predictions show the potential consequences of climate change on future human well-being and wildlife survival in these shared landscapes.

Our second main finding underlines that the future of human–wildlife coexistence in Central Asia will be shaped by region-specific patterns of conflict and competition, while opportunities for recovery and restoration also exist. Our models predict an increase in potential human–snow leopard conflict in Tajikistan, raising concerns about heightened depredation and retaliatory killings. This is particularly worrisome, as more favorable conditions for livestock pastoralism could be accompanied by a higher number of livestock per herd, potentially causing rangeland degradation. This risk is specifically high in the foothills, where livestock grazing is expected to intensify substantially. By contrast, Kyrgyzstan is expected to see a stable or a small decline in potential human–snow leopard conflict. While a reduction in overlap may appear to provide opportunities for lower conflict and thus coexistence (Xiao et al. 2022), the shrinkage of the snow leopard habitat in this landscape is concerning and casts doubt on the ability of the species to persist in the Kyrgyz study landscape in the future, particularly under more extreme climatic change.

Similarly, we predicted that the spatial overlap between wild ungulates and livestock will decline in Kyrgyzstan but remain stable in Tajikistan. This pattern suggests that pastoralists in Tajikistan may continue to share grazing areas with wild herbivores, necessitating pasture management strategies to prevent overgrazing and disease transmission. Meanwhile, stable snow leopard–prey overlap in both landscapes indicates that prey availability (when poaching or hunting effects are not intensified) is unlikely to be a major limiting factor for snow leopards. This suggests that the prey population may continue to support snow leopards but shifts in human behaviors toward wildlife could still influence predator–prey dynamics. It is worth mentioning that initiatives in promoting community-based conservancies and creating livelihood options for local people through trophy hunting (Tajikistan) and eco-tourism (Kyrgyzstan) have remarkably improved the wild ungulate status in our study landscapes (Michel and Rosen 2024; Zuther et al. 2024), indirectly improving the snow leopard’s persistence and the resilience of coexistence. However, perturbations such as socioeconomic or political shocks or climate change may weaken the stability of these states and lead to reduced prey availability, which is an additional threat to the snow leopard in these landscapes.

The resilience of human–wildlife coexistence depends on several components, including the ability of both people and wildlife to adapt to changing conditions (Carter and Linnell 2016), in ways that support the needs of people and wildlife alike (Gao and Yu). Our third main findings suggest that Tajikistan may face a worsening state of human–wildlife interactions, with potentially more conflict and transitions to even weaker coadaptation states not consistent with coexistence. In Kyrgyzstan, while the snow leopard habitat is expected to decline, the reduced overlap with livestock suggests that conflict intensity may decrease, potentially leading to more stable coexistence states. In either case, the ability of pastoralists to adapt to changing grazing conditions and live with the snow leopard and other wildlife will be crucial for coexistence. For instance, in the eastern Qinghai-Tibet Plateau, a combination of seasonal rotational herding, adaptive pasture allocation, and communal herding led to the redistribution of depredation risk in space and time, contributing to higher levels of sustainable coexistence (Gao and Yu 2025). However, pastoralists are classified as having the least influence while being among the most impacted by climate change in our study landscapes (Skrimizea et al. 2022).

Here, we explored spatial overlaps between a carnivore, its prey, and livestock, which allowed linking to the framework by Carter and Linnell (2023), exploring the coexistence states outlined there. We highlight that this is quite far from a full operationalization of this framework. Future operationalization of the Carter and Linnell (2023) framework should benefit from several complementary elements: (1) further inclusion of local stakeholders in jointly defining more context-specific coexistence states (Glikman et al. 2021); (2) identifying and testing of empirical indicators that capture meaningful social–ecological variables, such as human tolerance, wildlife adaptability, and institutional capacity, to define thresholds for transitions between coexistence states; and (3) the role of institutions and policies in the perception of coexistence in the local context, which includes equitable distribution of risks, benefits, and decision-making power (Gao and Yu 2025). Still, our analyses provide concrete starting points for managing toward states more aligned with coexistence. For instance, shared pastures, especially those consistently being predicted as areas of overlap across time steps and climate scenarios, should be targeted for management practices to reduce conflict (e.g., predator-proof corrals) and to avoid competition over forage and space, as well as the risk of disease transmission (e.g., spatiotemporal zonation for wild ungulates and livestock grazing). Conservation planning under climate- and land-use changes must, therefore, integrate both ecological and social resilience strategies to ensure sustainable coexistence in the future (Newsom et al. 2023).

Our study has direct implications for conservation interventions in Central Asia. In Tajikistan, where human–snow leopard conflict is expected to intensify, proactive conflict mitigation measures such as predator-proof corrals (Bijoor et al. 2021; Kachel et al. 2022) and compensation schemes (e.g., community-led livestock insurance) (Ravenelle and Nyhus 2017; Alexander et al. 2021) should be targeted in the emerging overlap areas we identified. Additionally, community-based conservation programs that engage local pastoralists in snow leopard conservation efforts could help reduce retaliatory killings and foster coexistence (Jackson 2012). In Kyrgyzstan, where climate may negatively impact livestock pastoralism, policies should support alternative livelihoods and sustainable grazing practices to mitigate potential socioeconomic impacts on pastoral communities. Moreover, the restoration of degraded pastures, rotational livestock grazing in remaining suitable pastures, and the establishment of buffer zones between core wildlife habitats and grazing areas could help prevent overgrazing in Kyrgyzstan.

Despite valuable insights provided by our models and predictions, several limitations should be acknowledged. First, our species distribution models rely on climate and environmental predictors but do not account for finer-scale ecological processes such as shifts in vegetation composition and productivity, plant phenology, prey dynamics, intraspecies competition, or behavioral adaptations (Forrest et al. 2012). Future studies incorporating finer-scale data (e.g., movement) could enhance predictions of species responses to climate change. Second, our projections assumed that anthropogenic variables such as infrastructure and land use, other than pastoralism, remain relatively stable, although infrastructure development (e.g., roads, mining) could alter the landscapes. Incorporating more spatially detailed socioeconomic (e.g., urbanization, changing consumption patterns) and cultural (e.g., changing attitudes) factors into our models would further improve our predictions but these data are unfortunately unavailable. Third, our assumption that an increase in range overlap will result in increased conflict may appear simplistic. However, this has been shown to be the case for the snow leopard (Aryal et al. 2014a, 2014b; Wang et al. 2024), other carnivore species (Rabaiotti et al. 2021), and more generally, wildlife species (Newsom et al. 2023). However, these assumptions may differ from the responses of people and wildlife to climate change and, consequently, the interactions between them. Therefore, our results should be treated as indicative of coexistence scenarios rather than predictive. Finally, while our corral-based grazing estimator provides a useful approximation of grazing pressure, we did not include information on livestock numbers and composition, limiting our ability to precisely estimate competition with wild ungulates. For instance, we cannot exclude the possibility that some corrals were used historically but are currently abandoned. However, livestock corrals play an important role as conflict hot spots and, therefore, are highly relevant to our assessment of human–snow leopard conflict.

The resilience of human–wildlife interactions will depend on proactive planning to mitigate the negative impacts that climate change might have and make use of the opportunities it might bring about. Our two study landscapes show that both are plausible under climate change, highlighting the need for and potential benefits of adaptive management. Accordingly, our study is timely for devising climate-smart conflict mitigation measures. By integrating ecological modeling and local knowledge, we identified critical areas where conservation efforts should be focused. This would strengthen the resilience of fragile high mountain systems to future climate- and land-use pressures, with conservation implications beyond the studied system. By addressing the challenges and opportunities presented by climate change, we can enhance the long-term sustainability of human–wildlife coexistence in these critical landscapes.

## Supplementary Information

Below is the link to the electronic supplementary material.

Supplementary file1 (PDF 3781 KB)

## Data Availability

The data that support the findings of this study are not openly available due to reasons of sensitivity and are available from the corresponding author upon reasonable request.

## Footnotes

1 https://earth.google.com
