# Supplementary material for: Snow leopards, prey, and pastoralists: Understanding the impacts of climate change on human–wildlife coexistence in Central Asia
Source: Ambio. 2025 Dec 7;55(5):1079–95. doi: 10.1007/s13280-025-02321-7 (PMC13035994; doi:10.1007/s13280-025-02321-7)
Supplement: Supplementary file 1 — Supplementary file1 (PDF 3781 KB) [file 13280_2025_2321_MOESM1_ESM.pdf]

Snow leopards, prey and pastoralists: understanding the impacts of climate change on human-wildlife coexistence in Central Asia

Ambio – *Special Issue: Human-wildlife relations: resilience thinking for coexistence*

Supplementary Information

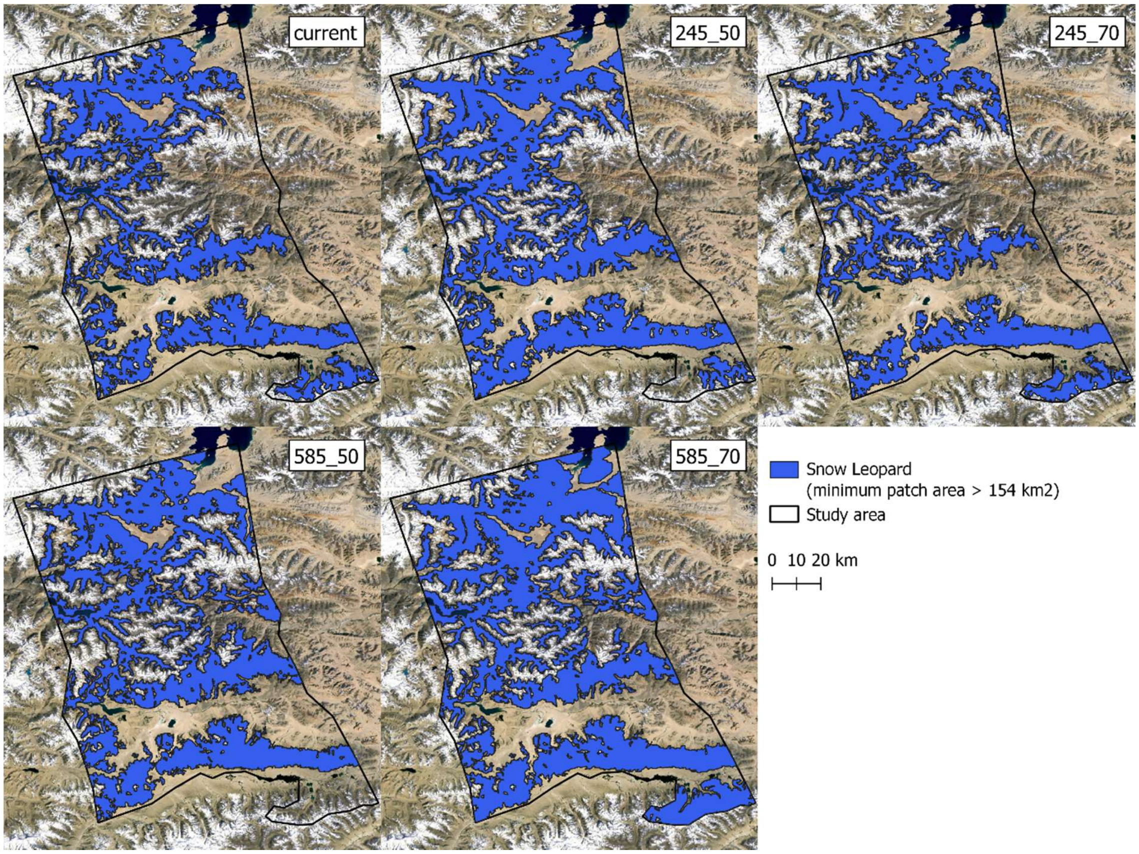

Figure S1. Predicted habitat of snow leopard in study landscape in Tajikistan under current and future climate scenarios (SSP2-4.5 and SSP5-8.5) and time steps (2050 and 2070) filtered for a minimum patch size of 154km².

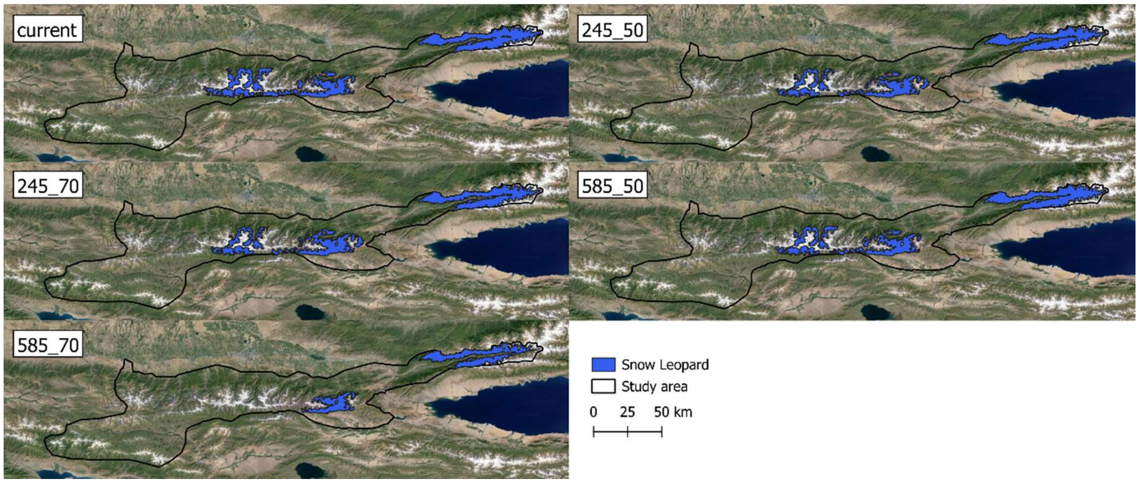

Figure S2. Predicted habitat of snow leopard in study landscape in Kyrgyzstan under current and future climate scenarios (SSP2-4.5 and SSP5-8.5) and time steps (2050 and 2070) filtered for a minimum patch size of 154km^2.

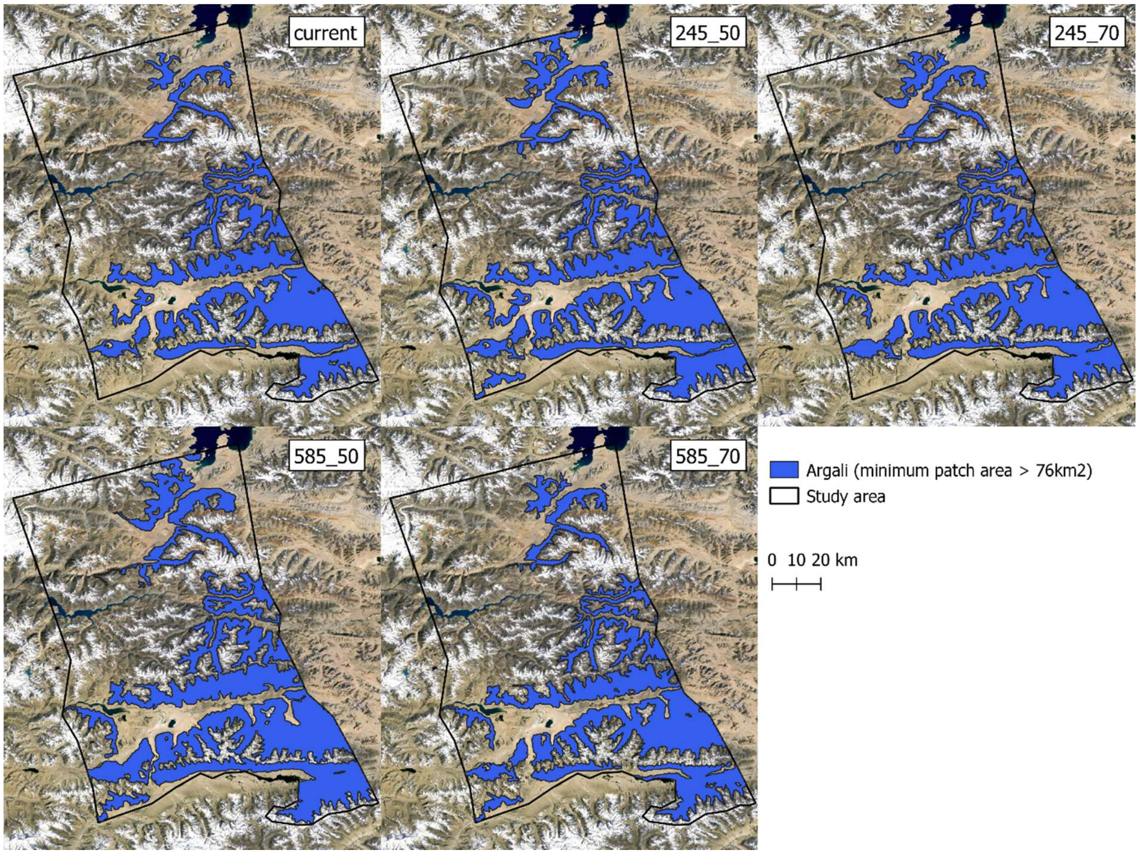

Figure S3. Predicted habitat of argali in study landscape in Tajikistan under current and future climate scenarios (SSP2-4.5 and SSP5-8.5) and time steps (2050 and 2070) filtered for a minimum patch size of 76km^2.

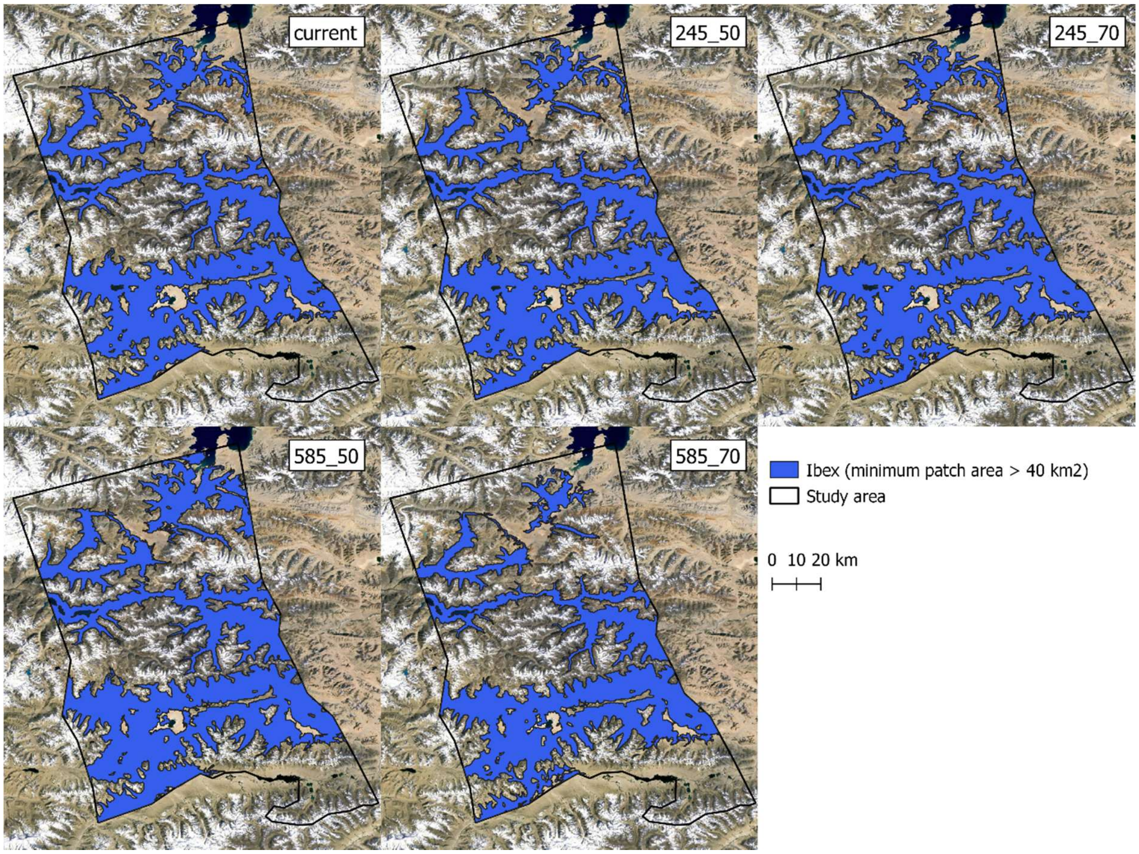

Figure S4. Predicted habitat of Asiatic ibex in study landscape in Tajikistan under current and future climate scenarios (SSP2-4.5 and SSP5-8.5) and time steps (2050 and 2070) filtered for a minimum patch size of 40km^2.

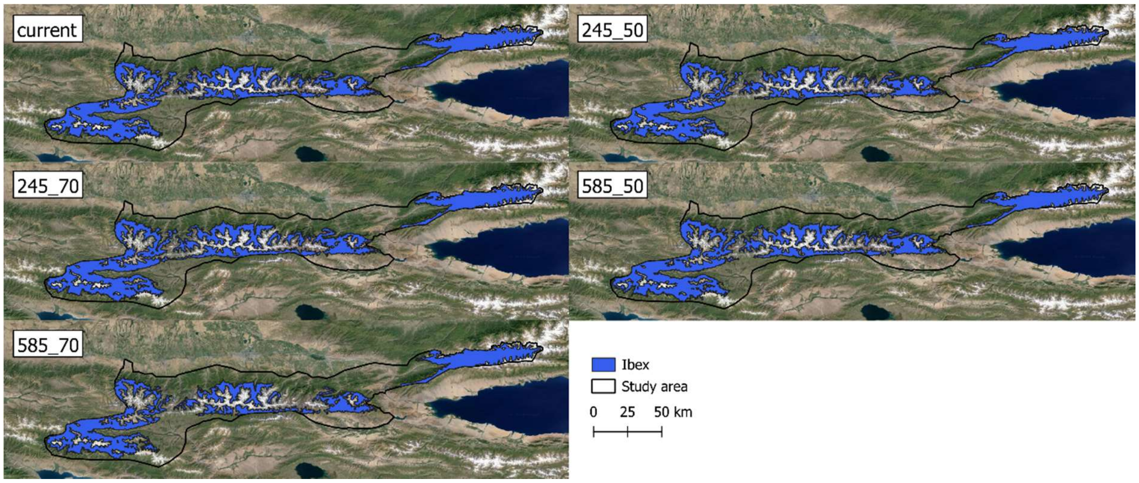

Figure S5. Predicted habitat of Asiatic ibex in study landscape in Kyrgyzstan under current and future climate scenarios (SSP2-4.5 and SSP5-8.5) and time steps (2050 and 2070) filtered for a minimum patch size of 40km^2.

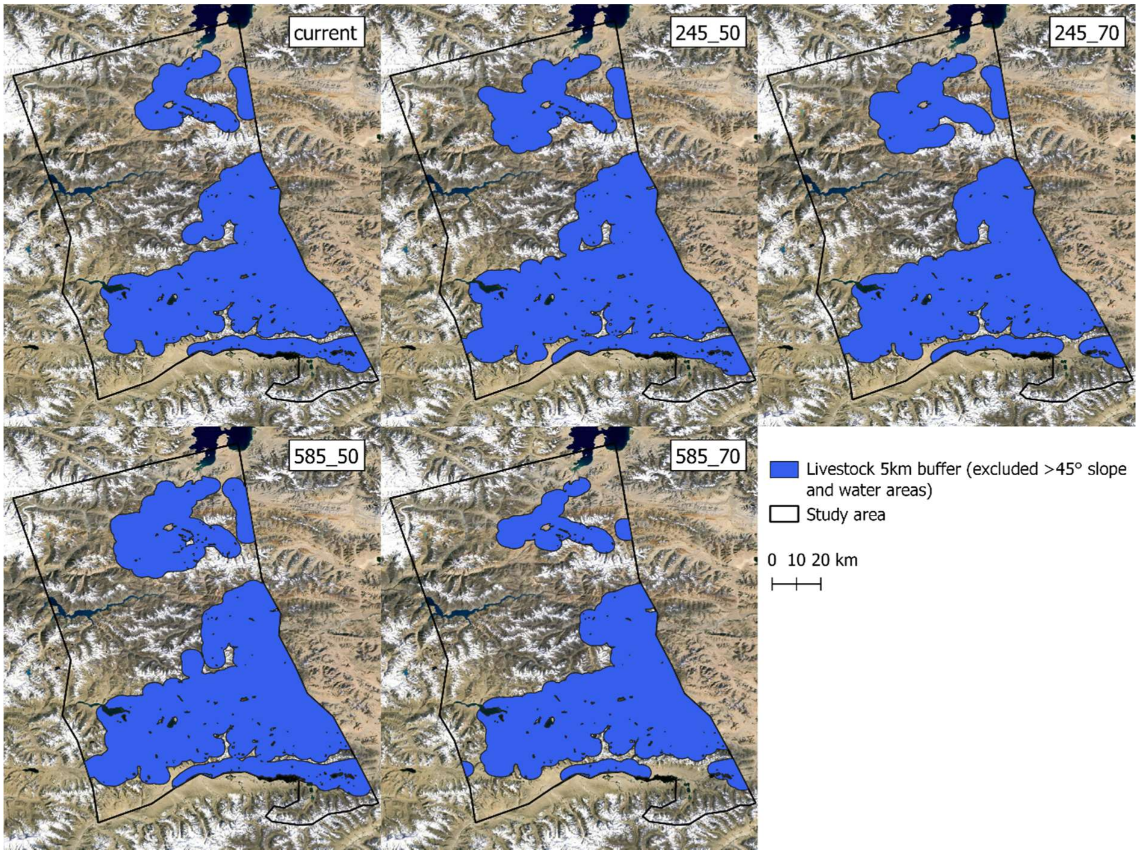

Figure S6. Predicted distribution of livestock pastoralism in study landscape in Tajikistan under current and future climate scenarios (SSP2-4.5 and SSP5-8.5) and time steps (2050 and 2070). A buffer of 5km was added to the identified patches to account for livestock movement around the corrals. Bodies of water and slopes >45 degrees were removed.

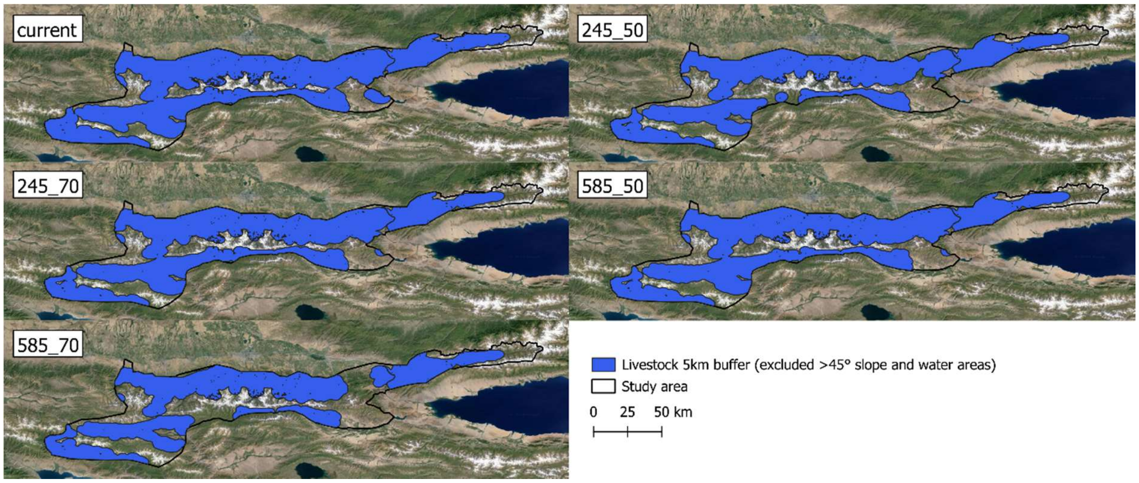

Figure S7. Predicted distribution of livestock pastoralism in study landscape in Kyrgyzstan under current and future climate scenarios (SSP2-4.5 and SSP5-8.5) and time steps (2050 and 2070). A buffer of 5km was added to the identified patches to account for livestock movement around the corrals. Bodies of water and slopes >45 degrees were removed.

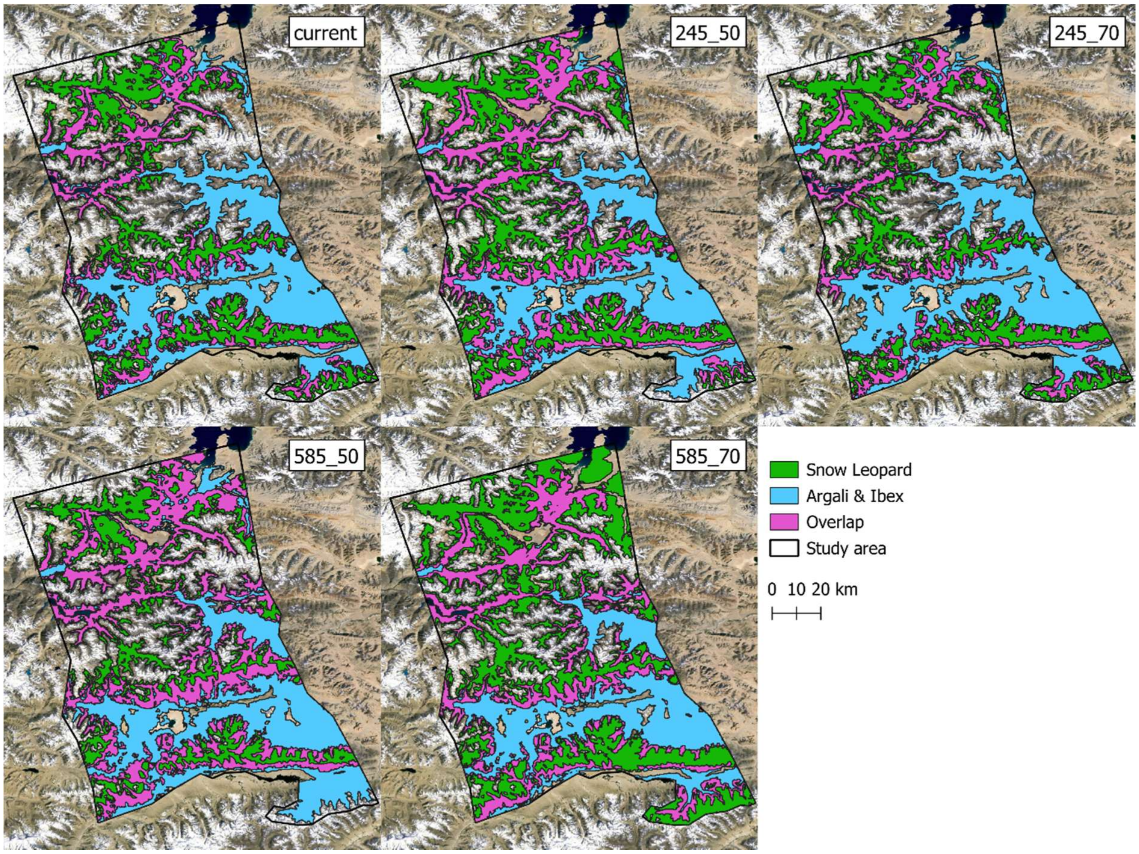

Figure S8. Current and future range overlap of the snow leopard-wild ungulate (argali and Asiatic ibex) in Tajikistan under different climate scenarios (SSP2-4.5 and SSP5-8.5) and time steps (2050 and 2070).

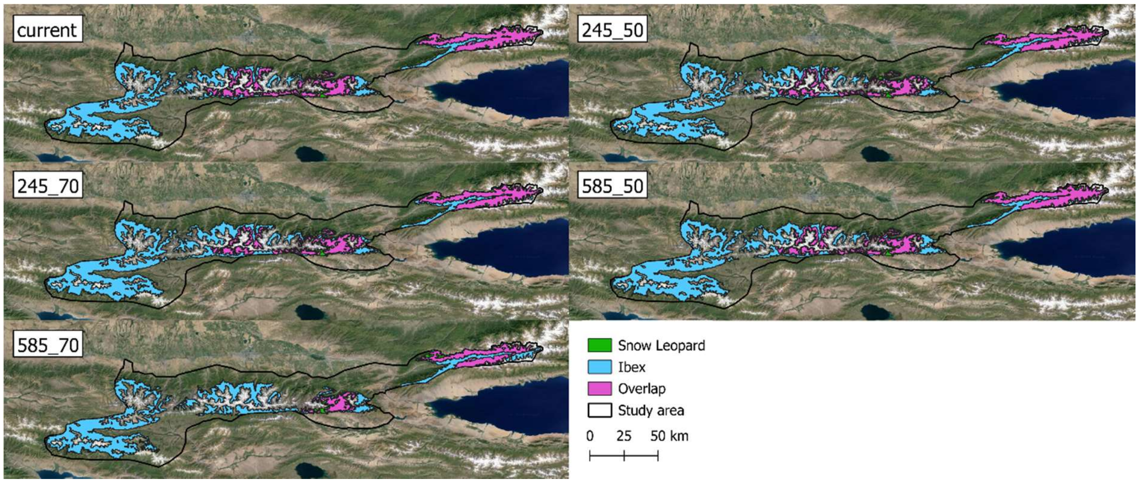

Figure S9. Current and future range overlap of the snow leopard-wild ungulate (Asiatic ibex) in Kyrgyzstan under different climate scenarios (SSP2-4.5 and SSP5-8.5) and time steps (2050 and 2070).

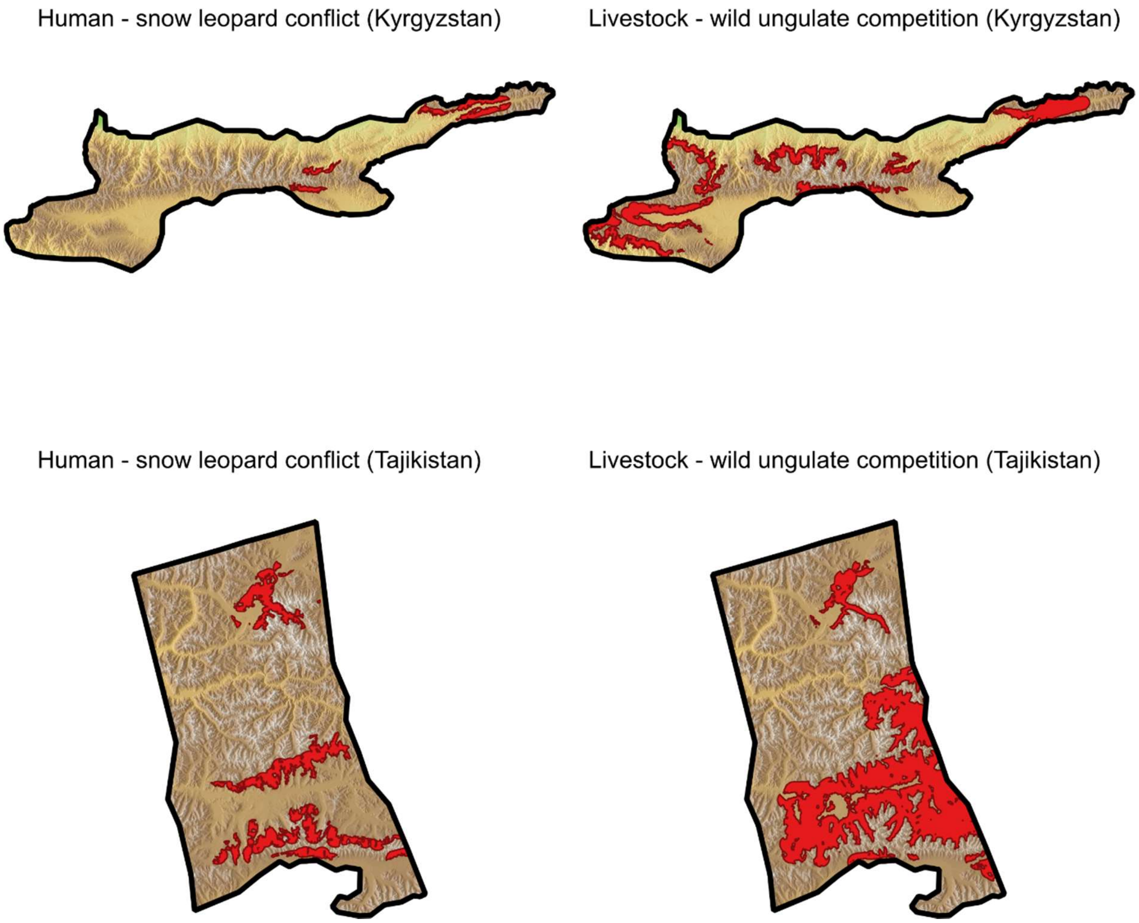

Figure S10. Distribution of potential human-snow leopard conflict (left) and livestock-wild ungulates competition (right) hotspots across climate scenarios (SSP2-4.5 and SSP5-8.5) and time steps (2050 and 2070) in Kyrgyzstan (top) and Tajikistan (bottom).

Table S1. The definitions of states of human-wildlife interactions consistent or not consistent with coexistence, following Carter and Linnell (2023).

State	Wildlife adaptation	Human adaptation	Definition
**Consistent with coexistence**			
Fragile stability	Weak	Weak	Wildlife and people ignore each other as their reciprocal negative impacts are negligible
Conservation reliance	Weak	Strong	Sustained actions taken to conserve species highly sensitive to human disturbance
Tolerant synanthropy	Strong	Weak	People tolerate the presence of synanthropic species (i.e., evolved to live near humans)
Sustained co-benefit	Strong	Strong	People and wildlife mutually benefit from the presence of each other
**Not consistent with coexistence**			
Zero sum losers	Weak	Weak	Wildlife populations decline proportional to increasing human disturbance
Eradication	Weak	Strong	Wildlife populations cannot recover from concerted eradication efforts by humans
Sporadic nuisance	Strong	Weak	Minor wildlife impacts occasionally warrant the management of nuisances
Reciprocal damages	Strong	Strong	Frequent encounters prompt strong responses that perpetuate negative impacts on both parties

Table S2. Key mediating factors for the allocation of the coexistence states in each country, based on the stakeholder workshops and technical reports (Skrimizea et al. 2022).

Category	Tajikistan	Kyrgyzstan
Governance and institutional capacity	Weak governance and institutional capacity: limited state support for pasture management or conservation enforcement; pasture-user associations play a minor role.	Moderate government capacity: functioning pasture committees supported by a legal framework, though capacity and enforcement vary by region.
Conservation funding	Wildlife conservation relies on limited short-term NGO/donor programs with uncertain long-term continuity. Conservancies with trophy hunting income support conservation on a limited scale.	NGO/donor program funding is more established but mostly focused on local livelihoods and eco-tourism programs, but with uncertain long-term continuity.

Livelihoods and economic diversification	High dependency on livestock for subsistence and income, with little economic diversification.	Livelihood diversification is limited in rural areas but eco-tourism and donor-supported conservation projects offer emerging alternatives.
Cultural values	The snow leopard has a high status in local culture but this often does not prevent persecution in cases of conflict.	Public and cultural value is placed on snow leopards, especially among youth and in areas with tourism potential (i.e., brings recreational and economic benefits).
Human dimensions of conflict	Low tolerance for snow leopard in some communities, particularly where depredation incidents occur without compensation; social norms may support retaliatory actions.	Depredation continues to cause resentment, especially where mitigation is lacking but retaliatory behaviour appears less prevalent than in Tajikistan.
Conflict mitigation measures	Absence of formal mitigation tools: no widespread compensation schemes, livestock insurance, or predator-proof corrals outside isolated NGO projects.	Ongoing pilot projects: introduction of predator-proof corrals and community-based monitoring in some areas, though not yet widely scaled.
Integration of wildlife in land use planning	Limited integration of wildlife in pasture or land-use planning, and minimal involvement of herders in formal conservation initiatives.	Wildlife considerations are not yet fully integrated into pasture management plans.
Adaptive capacity and perceptions	Stakeholder insights suggest a general sense of vulnerability and low adaptive capacity to ecological change.	Stakeholder inputs indicate growing awareness and interest in coexistence, but concern about institutional follow-through and long-term funding.
